# Temporal Dynamics of Bacterial Communities in a Pilot-Scale Vermireactor Fed with Distilled Grape Marc

**DOI:** 10.3390/microorganisms8050642

**Published:** 2020-04-28

**Authors:** María Gómez-Brandón, Manuel Aira, Natielo Santana, Marcos Pérez-Losada, Jorge Domínguez

**Affiliations:** 1 Grupo de Ecoloxía Animal (GEA), Universidade de Vigo, E-36310 Vigo, Spain; 2 Department of Soil Science, Federal University of Santa Maria, 97119-900 Santa Maria, Rio Grande do Sul, Brazil; 3 Computational Biology Institute, Department of Biostatistics and Bioinformatics, Milken Institute School of Public Health, George Washington University, Washington, DC 20052, USA; 4 CIBIO-InBIO, Centro de Investigação em Biodiversidade e Recursos Genéticos, Universidade do Porto, Campus Agrário de Vairão, 4485-661 Vairão, Portugal

**Keywords:** winery byproducts, steam distillation, earthworms, microbial communities, vermicompost, 16S rRNA, metagenomics

## Abstract

Vermicomposting has been found as a profitable approach to dispose of and treat large quantities of raw grape marc. However, less information is available with regard to its efficiency for treating distillery winery byproducts, even though distillation has been widely used as a way to economically valorize grape marc. As such, we sought to characterize the compositional and functional changes in bacterial communities during vermicomposting of distilled grape marc by using 16S rRNA high-throughput sequencing. Samples were collected at the initiation of vermicomposting and at days 14, 21, 28, 35 and 42. There were significant changes (*p* < 0.0001) in the bacterial community composition of distilled grape marc after 14 days of vermicomposting that were accompanied by twofold increases in bacterial richness and diversity from a taxonomic and phylogenetic perspective. This was followed by significant increases in functional diversity of the bacterial community, including metabolic capacity, lignin and cellulose metabolism, and salicylic acid synthesis. These findings indicate that the most striking compositional and functional bacterial community changes took place during the active phase of the process. They also pinpoint functional attributes that may be related to the potential beneficial effects of distilled grape marc vermicompost when applied on soil and plants.

## 1. Introduction

Grape marc, the major solid byproduct of the winemaking industry, has been used as an additive in animal feeding [1], and as a soil conditioner owing to its high level of organic matter and macronutrients [2,3,4,5,6,7]. This winery byproduct is also sent to distilleries, to recover ethanol for its further use in the elaboration of alcoholic beverages [8,9], and to cosmetic and pharmaceutical industries [6]. Moreover, grape marc can represent a valuable alternative to replace fossil fuels by means of bioethanol generation through distillation [10]. Altogether, it makes distillation a profitable way to economically valorize grape marc.

Nevertheless, the process of distillation also involves the generation of liquid and solid distillery effluents such as vinasses and distilled grape marc that must be treated, disposed of or reused properly to avoid negative environmental impacts [11]. As in raw grape marc, the low pH and the presence of phytotoxic and antimicrobial compounds make distilled grape marc a troublesome waste stream product, which, if not properly treated, can cause soil acidification, groundwater pollution and oxygen depletion in soil [12]. In this sense, stabilization of distilled grape marc via composting has been explored [12,13,14,15]. However, the low pH of distilled grape marc poses challenges for large-scale composting trials because it could inhibit the transition between mesophilic and thermophilic composting phases [13]. Co-composting of distilled grape marc with other organic materials like green waste has been shown as a more effective alternative improving not only the compost quality but also shortening the time required to achieve stabilization [14]. Vermicomposting has also been successfully used to dispose of and treat large quantities of raw grape marc [2,3,4,5,6,7,16,17]. On the one hand, it may provide a means to neutralize the pH of this winery byproduct, as previously shown by Domínguez et al. [2]. And, on the other hand, vermicomposting has been shown to effectively reduce organic biomass and generate high-quality fertilizer with beneficial effects on soil and plants [18,19].

Nonetheless, there is scarce information about the feasibility of vermicomposting for treating distillery residues [20]. Both raw and distilled grape marc are expected to differ in their autochthonous microbiota likely due to the strong selective pressures exerted by high ethanol concentrations, low pH, low oxygen levels and temperature fluctuations in the microbial composition of raw grape marc throughout the distillation process [21]. Such differences in the initial microbial composition of the starting materials may have important consequences for driving bacterial succession during the vermicomposting process [22]. The temporal shifts in microbial communities during vermicomposting can be seen as an example of heterotrophic ecological succession driven by changes in both the quantity and quality of available organic carbon sources. The early changes in the community composition are mainly represented by bacteria that have recently passed through the earthworm gut and been excreted [23]. These egested materials act as a source of microorganisms and nutrients, and their rapid decomposition will result in the release of labile nutrient pools supporting the growth of copiotrophic bacteria, which are characterized by faster rates of carbon turnover, at the earlier time points. As microbial succession progresses, copiotrophic groups will be replaced by oligotrophic bacteria with a higher substrate utilization efficiency and the ability to metabolize the remaining recalcitrant substrates in the casts during the maturation stage [24]. By using sequencing-based microbiome analyses it has enabled us to enhance the throughput, and phylogenetically group and name a larger number of bacterial taxa in the vermicompost microbiome, as recently described for raw grape marc-derived vermicomposts of the cultivars Albariño [25] and Mencía [7]. Moreover, the application of high-throughput sequencing approaches has also facilitated the identification of bacterial taxa that drive keystone functions that may explain the beneficial effects of vermicomposts when applied to the soil and plants [25,26].

However, it is not known yet if the bacterial communities involved in the various stages of vermicomposting of marcs derived from the distillation process will undergo similar compositional and functional changes as those reported for raw grape marc over the course of the process. Therefore, the aim of the present study was to characterize the taxonomic and functional diversity of the bacterial communities involved in the vermicomposting of distilled grape marc of *Vitis vinifera* L. cv. Albariño in a pilot-scale vermireactor by means of 16S rRNA high-throughput sequencing.

## 2. Materials and Methods

### 2.1. Distilled Grape Marc

We chose marc derived from distillation of *Vitis vinifera* cv. Albariño grapes since this grape variety represents 95% of the annual white grape harvest in northwestern Spain. The distilled grape marc was kindly supplied by a winery located in Pontevedra (Galicia, NW Spain). It was stored at 4 °C until needed and turned and moistened with water during two days prior to the vermicomposting trial.

### 2.2. Vermicomposting Set-Up and Sampling Design

The distilled grape marc was processed in a rectangular metal pilot-scale vermireactor (4 m long × 1.5 m wide × 1 m high) housed in a greenhouse with no temperature control over a period of 42 days. The vermireactor set-up has been previously described by Kolbe et al. [25]. The population density of earthworms (*Eisenia andrei*) was close to its maximum capacity, having a value of 10,923 ± 1783 individuals m^−2^ that corresponds to 289 ± 72 mature earthworms m^−2^ and 10,634 ± 1767 immatures m^−2^, with a mean biomass of 1704 ± 251 g m^−2^. The earthworm population density in the present study was 36 times higher when compared to that in Kolbe et al. [25]. As a consequence, the vermicomposting time was shortened by half in the current study and the distilled grape marc was completely processed by the earthworms in 42 days.

For the characterization of the molecular and the microbial properties, the distilled grape marc layer (12 cm height) was divided into five sections; five samples (10 g) were taken at random from each section at day 0 (fresh distilled grape marc) and after 14, 21, 28, 35 and 42 days of vermicomposting. Samples were bulked and stored in plastic bags at −80 °C until needed.

### 2.3. Microbial Activity

Microbial activity was assessed as basal respiration by measuring the rate of evolution of CO_2_ after 6 h of incubation. The evolved CO_2_ was trapped in NaOH and then measured by titration with HCl to a phenolphthalein endpoint after adding excess BaCl_2_ [27].

### 2.4. DNA Sequencing and Bioinformatic Analyses

DNA was extracted from 0.25 g (fresh weight, fw) of distilled grape marc using the MO-BIO PowerSoil^®^ kit (MoBio Laboratories Inc., Carlsbad, California) according to manufacturer’s protocols. DNA quality and quantity were determined using BioTek’s Take3™ Multi-Volume Plate (Sinergy^TM^ 2 Multi-Mode Microplate Reader, Bio-Tek Instruments, Inc.), as previously described by Kolbe et al. [25]. In order to describe the bacterial changes during vermicomposting of distilled grape marc, we focused on a fragment of the 16S rRNA gene covering the V4 region, by using a dual-index sequencing strategy, as described by Kozich et al. [28]. In total, 30 DNA samples representing different sampling times (0, 14, 21, 28, 35 and 42 days) were sequenced using the Illumina MiSeq platform at the Center for Michigan System, University of Michigan.

The DADA2 pipeline (version 1.12) was used to infer the amplicon sequence variants (ASVs) present in each sample [29]. Standard filtering parameters were used, with forward and reverse reads truncated at 200 nt and 120 nt respectively, and with a maximum of two expected errors per read. Default settings were used for ASV inference and chimera detection. Taxonomic assignment was performed against the Silva v132 database using the *assignTaxonomy* function in DADA2, which implements the RDP naive Bayesian classifier [30,31]. A minimum bootstrap confidence of 80 was used for assigning taxonomy. A total of 1,408,391 sequences passed all quality filters and were assigned to 3410 ASVs. Sequence data have been uploaded to the Sequence Read Archive database under accession PRJNA602410.

The functional composition of the metagenomes was predicted using the Phylogenetic Investigation of Communities by Reconstruction of Unobserved States software package (PICRUSt) [32]. Predicted metagenomes were collapsed using the Kyoto Encyclopedia of Genes and Genomes (KEGG) pathway metadata [33]. Metagenome functional contributions were partitioned according to function, operational taxonomic unit (OTU), and sampled to evaluate how the OTUs responsible for specific functional roles change during vermicomposting of distilled grape marc.

ASVs with less than three sequences and not present in at least 5% of the samples were removed, thus eliminating 59.88% of the ASVs but only 1.85% of the sequences. Rarefaction curves indicated that the sampling depth was optimal for all samples in the full data set (3410 ASVs and 1,408,391 sequences, Supplementary Figure S1) and the filtered data set (1368 ASVs and 1,382,365 sequences, Supplementary Figure S2). We normalized ASV counts using the variance-stabilizing transformation for analysis that assume homoscedasticity or could be influenced by unequal variances [34]. When analysing differential ASV abundances we normalized raw ASV counts using negative binomial models as implemented in the package DESeq2 [34,35]. Differential abundances of ASVs and other bacterial taxa (phylum and class) were determined according to Wald tests and *p*-values adjusted by false discovery rate (FDR < 0.05). Because multiple pairwise Wald tests were conducted for each pairwise time comparison (0–14, 14–21, 21–28, 28–35 and 35–42 days), we further adjusted these *p*-values using the Benjamini–Hochberg method to correct for multiple testing. After correction, nonsignificant contrasts were considered to have an effect size (log2-fold change) of zero.

We defined the core microbiome of vermicomposting of the distilled grape marc as that comprised of ASVs present in all of the samples processed by earthworms (samples day 14 to day 42). We decided not to consider the fresh distilled grape marc because it is not an “earthworm-processed material”. An approximate maximum-likelihood phylogenetic tree was inferred using FastTree 2.1 [36]. Taxonomic α-diversity was calculated as the number of observed ASVs, and diversity and richness were estimated with the Shannon and Chao1 indexes, respectively. Phylogenetic diversity was calculated as Faith’s phylogenetic diversity [37]. Taxonomic β-diversity at the ASV level was estimated as the difference in composition of the bacterial taxonomic community between samples from different times during vermicomposting. This was done by coupling principal coordinate analysis (PCoA) with distance matrices that take the abundance of ASVs into account (Bray–Curtis) or not (Jaccard). Phylogenetic β-diversity was also estimated by PCoA of weighted (considering abundance of ASVs) and unweighted UniFrac matrix distances [38] by using the phyloseq library [34]. Mixed linear effect models were applied using the ‘nlme’ R package [39] to evaluate the effect of time on α- and β-diversity (PCoA scores) of the distilled grape marc bacterial communities. Time was the fixed factor and the effect of time nested in each sample was considered as a random factor to account for nonindependence of samples due to repeated measures. The normality of residuals and homogeneity of variance across groups was checked for each variable. The Tukey and Benjamini–Hochberg FDR tests, as implemented in the R package ‘multcomp’ [40], were used for post hoc comparisons and for multiple test corrections, respectively. Venn diagrams were constructed using the R package Vennerable [41] in order to show the number of ASVs exclusive and shared between raw and distilled grape marc samples. All analyses were performed in R version 3.5 [42], while all figures were created using the R package ggplot2 [43].

## 3. Results

### 3.1. Changes in Microbial Activity during Vermicomposting of Distilled Grape Marc

**Figure 1:**
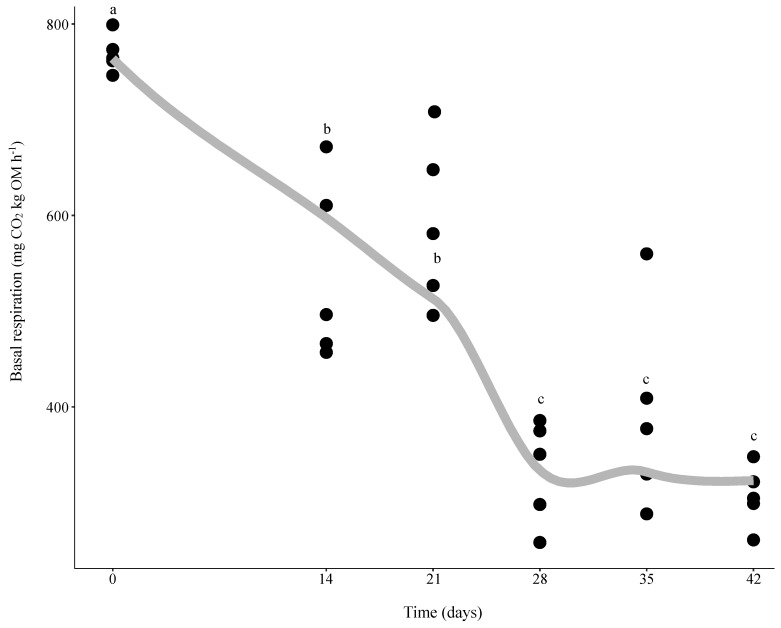
Changes in microbial activity assessed as basal respiration during vermicomposting of distilled grape marc derived from the white winemaking process of the grape variety Albariño. Individual values (*n* = 5) are plotted for each time point and the curve was plotted using the “loess” smoothing method in ggplot2 [43].

Microbial activity assessed as basal respiration significantly decreased from the beginning of the experiment (day 0) until day 28, followed by no more noticeable changes until the end of the trial on day 42 (Figure 1). This points to the effectiveness of vermicomposting at biologically stabilizing the distilled grape marc, as shown by the lower and stable values of basal respiration from day 28 onwards (Figure 1). Moreover, these results indicate that the contributions of earthworms to the process can be grouped into two stages, comprising the active phase of vermicomposting (from day 0 to day 28) and the maturation (from day 28 to day 42) phase.

### 3.2. Changes in Bacterial Community Composition during Vermicomposting of Distilled Grape Marc

**Figure 2:**
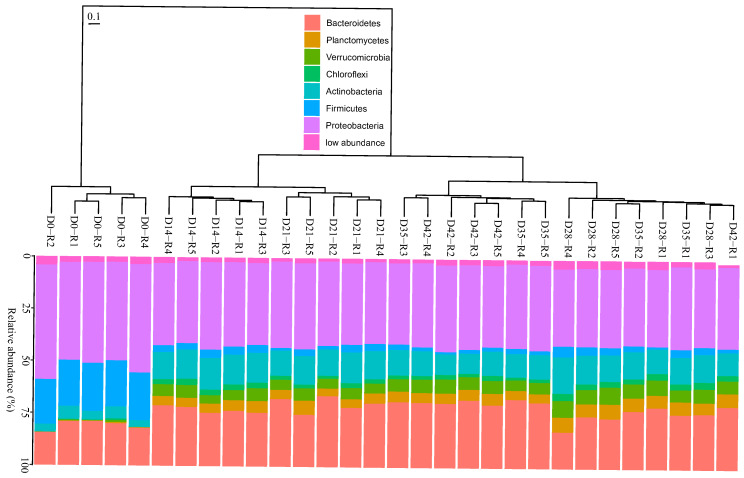
Changes in composition of the bacterial communities (phylum level) during vermicomposting of distilled grape marc derived from the white winemaking process of the grape variety Albariño. The dendrogram represents the dissimilarity of bacterial communities at the amplicon sequence variant (ASV) level (unweighted UniFrac distances, Ward method). Bars represent the relative abundance of most abundant bacterial phyla. Low abundance bacterial phyla (< 1%) were grouped together.

**Figure 3:**
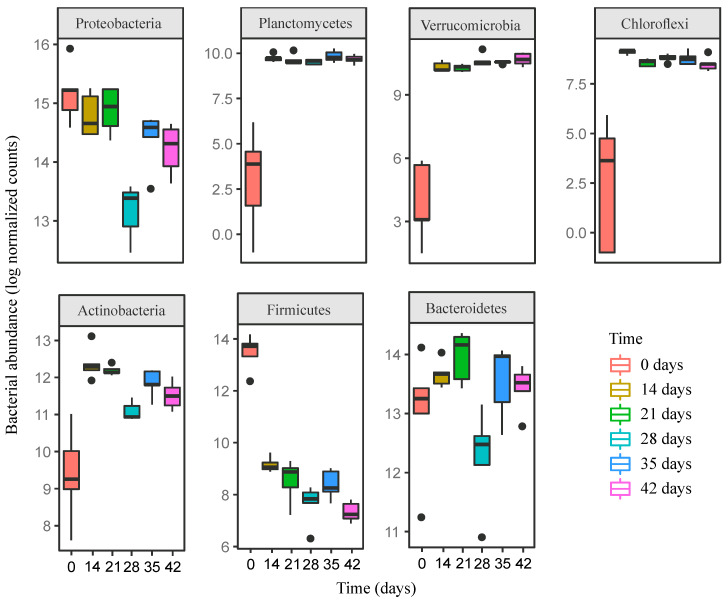
Boxplots showing the changes in the normalized abundance of the most abundant bacterial phyla during vermicomposting of distilled grape marc derived from the white winemaking process of the grape variety Albariño. Abundance changes are expressed as log2-fold changes.

Proteobacteria accounted for the most significant proportion of bacterial communities, nearly 50% of the sequences, in the fresh distilled grape marc (Figure 2). They continued to dominate the bacterial microbiome when the initial substrate was processed by the earthworms after 14 days of vermicomposting (Figure 2), although later on (days 21 and 28) they experienced a significant reduction in differential abundance (Figure 3). This was followed by a significant increase in their abundance between days 28 and 35, reaching similar levels to those in the beginning, and no more noticeable changes were recorded until the end of the trial (Figure 3). The three proteobacterial classes (*Gamma*-, *Alpha*-, and *Deltaproteobacteria*) followed distinct trends over the course of the process (Supplementary Figure S3).

In addition to Proteobacteria, ASVs from the phyla Firmicutes, Bacteroidetes and Actinobacteria, with minor contributions of the phyla Planctomycetes, Verrucomicrobia and Chloroflexi, made up about another half of the sequences of the fresh distilled grape marc (Figure 2). Earthworms significantly reduced the abundance of Firmicutes within the first 14 days of vermicomposting followed by no more noticeable differences until day 35 (Figure 2 and Figure 3), after which its abundance was reduced between this time point and day 42 (Figure 3). A similar trend was observed for the classes *Clostridia* and *Negativicutes* throughout the process of vermicomposting (Supplementary Figure S3). In contrast, the class *Bacilli* barely changed in terms of abundance over the course of the process (Supplementary Figure S3). The phylum Bacteroidetes and in particular the class *Bacteroidia* showed similar values in terms of differential abundance until day 21, followed by a pronounced reduction between days 21 and 28 and a subsequent increase between days 28 and 35 (Figure 3 and Supplementary Figure S3).

For the phylum Actinobacteria and those that appeared in lower abundance (Planctomycetes, Verrucomicrobia and Chloroflexi), earthworms led to an increase in its differential abundance after 14 days of vermicomposting (Figure 3). This increase also held true for the respective bacterial classes on day 14 (Supplementary Figure S3). Later on, Actinobacteria experienced a reduction in their abundance between days 21 and 28 (Figure 3), while the abundance of the other three bacterial phyla remained without noticeable changes from day 14 onwards (Figure 3 and Supplementary Figure S3).

### 3.3. Changes in α- and β-Diversity during Vermicomposting of Distilled Grape Marc

**Figure 4:**
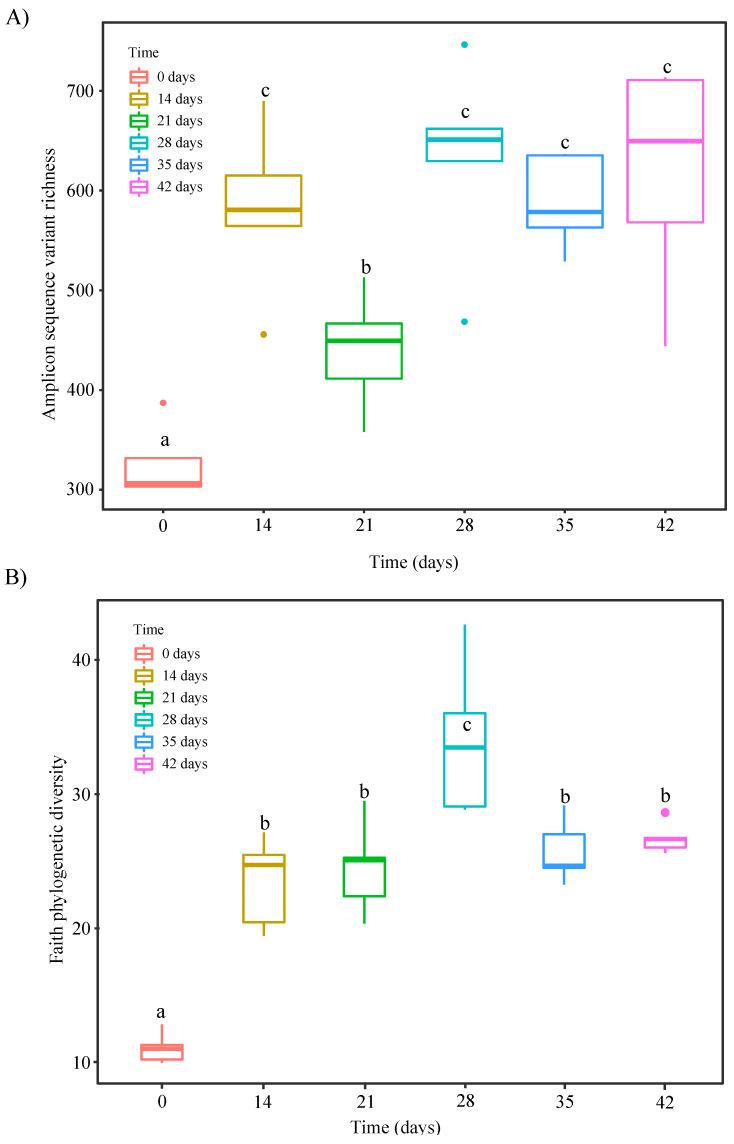
Changes in bacterial α-diversity during vermicomposting of distilled grape marc derived from the white winemaking process of the grape variety Albariño: (**A**) observed amplicon sequence variant (ASV) richness and (**B**) faith phylogenetic diversity. Different letters indicate significant differences between the different stages of the vermicomposting process (Tukey Honestly Significance Difference (HSD ) test, false discovery rate (FDR) corrected).

Bacterial alpha-diversity assessed as both ASV richness (Figure 4A) and Chao1 richness (Supplementary Figure S4A) was about two times higher after 14 days of vermicomposting than in the fresh distilled grape marc (day 0, Table 1). A significant increase on day 14 was also observed for both Shannon diversity (Figure S4B and Table 1) and Faith phylogenetic diversity (Figure 4B and Table 1). However, while lower values in ASV and Chao1 richness were recorded on day 21 compared to day 14 (Figure 4A, Supplementary Figure S4A; Table 1), this reduction was not observed in Faith phylogenetic diversity (Figure 4B) and Shannon diversity (Supplementary Figure S4B). Afterwards, there was a significant increase in bacterial α-diversity, from a taxonomic and phylogenetic viewpoint, between days 21 and 28 (Figure 4A,B and Supplementary Figure S4A,B), and no more noticeable changes were reported for the duration of the trial from a taxonomic perspective (Figure 4A and Supplementary Figure S4A,B). On the contrary, the phylogenetic diversity of bacterial communities was significantly reduced on the later time points (days 35 and 42), reaching similar levels than those reported for days 14 and 21 (Figure 4B).

**Figure 5:**
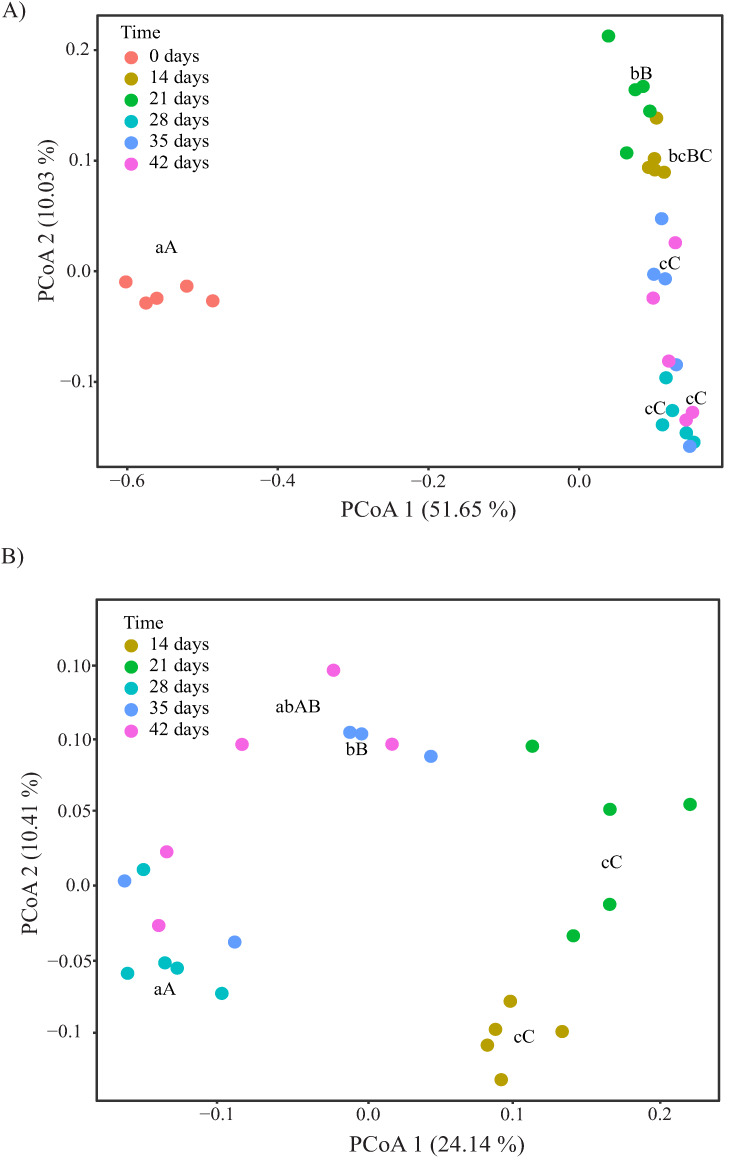
Changes in bacterial β-diversity during vermicomposting of distilled grape marc derived from the white winemaking process of the grape variety Albariño: (**A**) principal coordinate analysis (PCoA) of unweighted UniFrac including days 0, 14, 21, 28, 35 and 42; (**B**) PCoA of unweighted UniFrac across days 14, 21, 28, 35 and 42. Different capital and lower case letters indicate significant pairwise differences between the different stages of the vermicomposting process in PCoA1 and PCoA2 scores, respectively (Tukey HSD test, FDR corrected).

Principal coordinate analysis using unweighted UniFrac distances showed a clear separation between day 0 (fresh distilled grape marc) and days 14–42 (vermicompost samples) along the first dimension (PCoA1), which accounted for 51.65% of the total variance (Figure 5A). The second dimension (PCoA2), which explained 10.03% of the variance, mainly reflected the changes in bacterial community composition between the different stages of the vermicomposting process (Figure 5A). The 14- and 21-day samples (i.e., active phase) clustered together and separately from the middle (28 days) and later time points (35 and 42 days, maturation phase). A similar grouping was observed across the days 14, 21, 28, 35 and 42, when day 0 was excluded from the analysis (Figure 5B). These trends along PCoA1 and PCoA2 also held true using Bray–Curtis and Jaccard distance matrices, regardless of whether or not day 0 was included (Supplementary Figure S5A,C; Supplementary Figure S5D,F). However, the UniFrac distances showed no significant differentiation among the vermicompost samples along the second principal component (Supplementary Figure S5B,E).

### 3.4. Core bacterial Microbiome during Vermicomposting of Distilled Grape Marc

**Figure 6:**
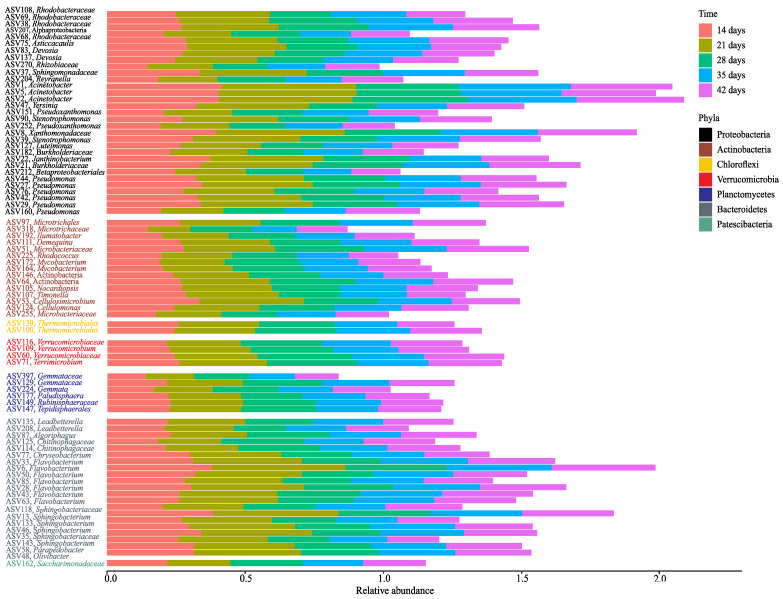
Relative abundance (%) of ASVs (phylum and genus or most inclusive taxonomy found) from the core microbiome of vermicomposting of distilled grape marc derived from the white winemaking process of the grape variety Albariño across days 14, 21, 28, 35 and 42. The initial substrate was not considered for determination of the core microbiome.

A total of eighty ASVs were identified as the bacterial core microbiome during vermicomposting of distilled grape marc derived from the grape variety Albariño. These ASVs were present in all of the samples from days 14, 21, 28, 35 and 42 (Figure 6), and represented 1.23% of the total ASVs and 8.20% of all sequences. The phyla Proteobacteria (31 ASVs) and Bacteroidetes (21 ASVs) contributed a higher number of ASVs to the core microbiome (Figure 6). The phylum Proteobacteria comprised ASVs from the families *Rhodobacteraceae*, *Rhizobiaceae*, *Sphingomonadaceae* and *Burkholderiaceae*, and from the genera *Devosia*, *Acinetobacter*, *Yersinia*, *Pseudoxanthomonas*, *Stenotrophomonas* and *Pseudomonas*, among others (Figure 6). The phylum Bacteroidetes was mainly represented by ASVs of the genera *Flavobacterium* and *Shingobacterium* (Figure 6). Other ASVs present in the core microbiome belonged to the phyla Actinobacteria (15 ASVs), Planctomycetes (six ASVs), Verrucomicrobia (four ASVs), Chloroflexi (two ASVs from the order *Thermomicrobiales*), and Patescibacteria (one ASV from the family *Saccharimonadaceae*) (Figure 6).

### 3.5. Functional Diversity of Bacterial Communities during Vermicomposting of Distilled Grape Marc

**Figure 7:**
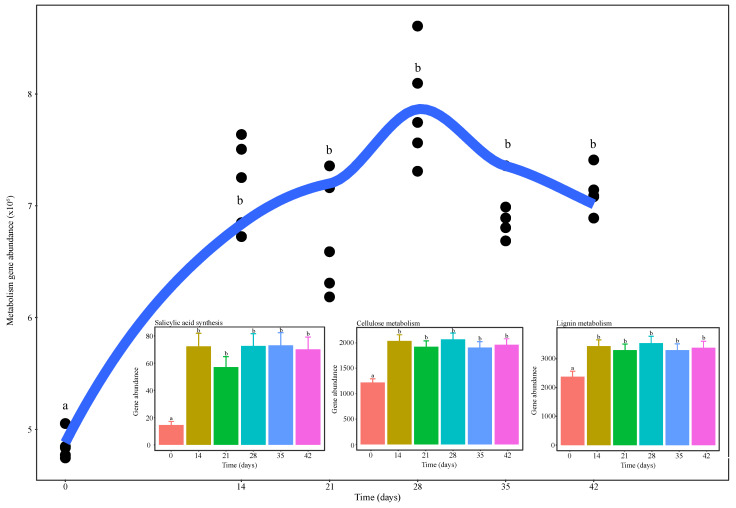
Changes in gene abundance of Kyoto Encyclopedia of Genes and Genomes (KEGG) orthologies predicted by the PICRUSt software and classified as “metabolism” in KEGG functional hierarchies during vermicomposting of distilled grape marc derived from the white winemaking process of the grape variety Albariño. Individual values (*n* = 5) are plotted for each time point and the curve was plotted using the “loess” smoothing method in ggplot2. The insets show changes in gene abundance of all PICRUSt-predicted enzyme-level genes for cellulose and lignin metabolism, and synthesis of salicylic acid. Values are presented as means ± standard error (*n* = 5).

Our PICRUSt analysis showed a significant increase in genes classified only as “metabolism” in KEGG functional hierarchies after 14 days of vermicomposting, followed by no more noticeable changes until the end of the trial (Figure 7). The same trend was reported for specific genes related to cellulose and lignin metabolism (F_20,5_ = 7.95, *p* = 0.0003; F_20,5_ = 3.86, *p* = 0.013 respectively; Figure 7 inset), and those involved in the synthesis of salicylic acid (F_20,5_ = 7.77, *p* = 0.0003; Figure 7 inset).

### 3.6. Comparison between Raw and Distilled Grape Marc Bacterial Community Composition and Diversity

The bacterial richness of distilled grape marc, assessed as ASV and Chao1 richness, was about three times higher than that in the raw grape marc at the beginning of their respective vermicomposting trials (Table 2). The same pattern was observed for bacterial diversity estimated by the Shannon index, which was twofold higher in the distilled grape marc on day 0 (Table 2). At the end of the process, both the distilled and raw grape marcs reached similar levels in terms of bacterial richness and diversity from a taxonomic viewpoint (Table 2), while the Faith phylogenetic diversity was about two times greater in the raw grape marc (Table 2).

When we compared with the raw grape marc bacterial community composition (Table 2), the relative abundance of Proteobacteria was similar to that of distilled grape marc at the beginning of the trial (day 0). However, Firmicutes showed a higher relative abundance in the raw than in the distilled grape marc (Table 2), while the opposite trend was reported for the phyla Bacteroidetes, Actinobacteria and Verrucomicrobia (Table 2). In addition, the phyla Planctomycetes and Chloroflexi appeared as dominant bacterial phyla (>1%) in the fresh distilled grape marc, but this was not the case for the raw grape marc (Table 2). At the end of the vermicomposting trial (day 91 for raw and day 42 for distilled), similar relative abundances of Proteobacteria, Firmicutes, Bacteroidetes, Actinobacteria and Verrucomicrobia were reported for the two winery byproducts (Table 2).

**Figure 8:**
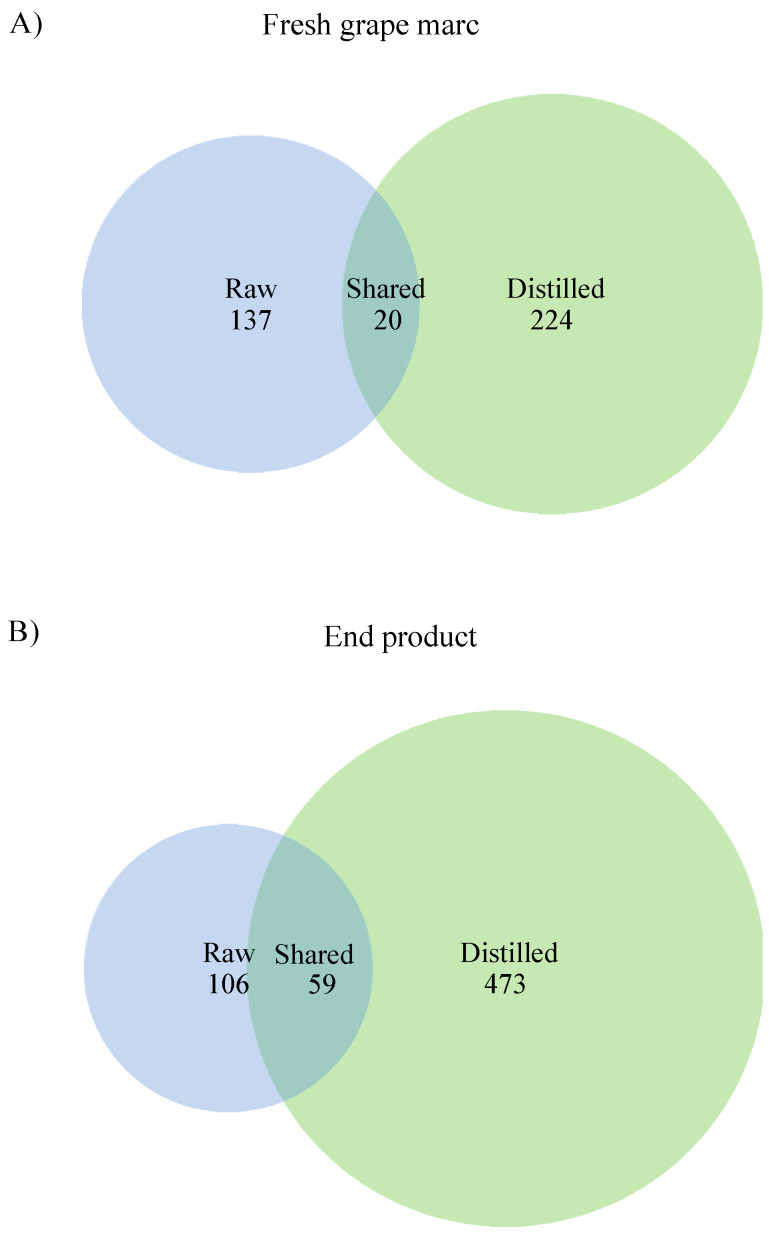
Venn diagrams showing the shared and exclusive ASVs between raw and distilled grape marc at the (**A**) beginning (day 0), and (**B**) end of the respective vermicomposting trials (days 91 and 42 for raw and distilled grape marc, respectively). The raw grape marc data were taken from Kolbe et al. [25].

Based on Venn diagrams twenty ASVs (5% of the total ASVs) were shared by both winery byproducts on day 0 (Figure 8A, Table S1), while 137 and 224 ASVs were exclusively found in raw and distilled grape marc, respectively (Figure 8A). When we compared the end products of the respective vermicomposting trials, raw and distilled grape marc shared 9% of the ASVs (Figure 8B). They had 59 ASVs in common (Figure 8B, Table S2), with a total of 106 and 473 ASVs being exclusive of raw and distilled grape marc derived vermicomposts, respectively (Figure 8B).

## 4. Discussion

Vermicomposting of distilled grape marc of the white grapevine cultivar Albariño was characterized by rapid bacterial community compositional changes. After 14 days of vermicomposting, the most striking shift at the phylum level involved a decrease in the abundance of Firmicutes (Figure 2 and Figure 3), as previously reported by Kolbe et al. [25] after only seven days of vermicomposting of raw grape marc obtained from the same grape variety. However, Firmicutes showed similar values in terms of abundance at the earlier time points before decreasing significantly on day 28 in the case of raw grape marc derived from the red grapevine cultivar Mencía [7]. This phylum is considered a fast-growing copiotroph group, while oligotrophic groups such as Actinobacteria, Planctomycetes, Verrucomicrobia and Chloroflexi reached higher levels on day 14, and the four of them, except for Actinobacteria, remained unchanged for the duration of the trial with distilled grape marc (Figure 3). Similarly, a rapid increase in Actinobacteria was found by Kolbe et al. [25] on day 7, while Gómez-Brandón et al. [7] observed a significant reduction in its abundance on day 14. Oligotrophic and copiotrophic bacterial groups mainly differed with regard to their growth strategies under nutrient rich conditions and their efficiency in metabolizing carbon substrates, i.e., labile and recalcitrant compounds for copiotrophs and oligotrophs, respectively [44]. Although 14 days is a rather short time, earthworms are known to accelerate the rate of organic matter decomposition (Figure 1; Domínguez et al. [45]), and consequently alter microbial community in such short timeframes [46]. These early changes in the composition of bacterial communities also held true at class level for the phyla Firmicutes, Actinobacteria, Planctomycetes, Verrucomicrobia and Chloroflexi (Figure S3). However, for Proteobacteria, the different bacterial classes displayed distinct patterns after 14 days of vermicomposting (Figure S3), probably due to the fact that this phylum is considered the most diverse and include a mix of taxa with different physiological strategies [47].

The phylum Bacteroidetes is associated with copiotrophic environments [48], but unlike Firmicutes its abundance was not reduced after 14 days of vermicomposting (Figure 3). A similar trend was reported by Kolbe et al. [25] and Gómez-Brandón et al. [7]. Indeed, Kolbe et al. [25] reported an increase in Bacteroidetes within the first 7 days of vermicomposting before decreasing slightly at the end of the process. This could be due to the fact that Bacteroidetes mostly comprise Gram-negative (G−) bacteria and previous findings based on phospholipid fatty acid (PLFA) analysis has shown that the passage of organic material through the gut of *E. andrei* reduced the abundance of Gram-positive (G+) bacteria to a greater extent than that of G− bacteria [46,49]. This reasoning was consistent with an increase in the abundance of the other G− bacterial phyla including Planctomycetes, Verrucomicrobia and Chloroflexi on day 14 (Figure 3). However, in the case of G+ bacterial phyla, we observed contrasting trends between the phyla Firmicutes and Actinobacteria after 14 days of vermicomposting (Figure 3). While the abundance of Firmicutes was reduced on day 14, Actinobacteria reached higher values at this time point compared to the fresh distilled grape marc (Figure 3). This discrepancy could be related to the ability of spore formation in Actinobacteria, which might make them more resistant to earthworm gut passage. Indeed, Aira et al. [23] observed that the passage of organic material through the gut of *E. andrei* increased the Actinobacteria abundance independently of the initial feedstock.

The rapid bacterial compositional changes were accompanied by an increase in richness and both taxonomic and phylogenetic diversity of distilled grape marc within the first 14 days of vermicomposting (Figure 4; Figure S4), as earlier shown by Kolbe et al. [25]. Gómez-Brandón et al. [7] recorded such an increase in bacterial alpha-diversity later on, on day 28. Similar findings were found for other parent materials in which epigeic earthworms led to an increase of bacterial diversity during the first stages of vermicomposting [26,50,51,52]. While steady and significant increases in bacterial richness and diversity of raw grape marc were reported for the duration of the vermicomposting trial by Kolbe et al. [25] and Gómez-Brandón et al. [7], we observed a reduction in these measurements in the case of distilled grape marc on day 21 (Figure 4A). This decrease was not reflected when phylogenetic information was incorporated (Figure 4B). This could be due to the fact that such a reduction in taxonomically diversity at this time point affected phylogenetically related taxa that perform complementary functions. At the end of the respective vermicomposting trials, similar levels of bacterial richness and diversity were reached in both the raw and the distilled grape marc from the white grape variety Albariño (Kolbe et al. [25] and this study; Table 2). These values were also close to those reported by Gómez-Brandón et al. [7] at the end of vermicomposting with Mencía raw grape marc. Taken together, this indicates that the bacterial community present in the raw and distilled grape marcs was largely affected and modulated by the earthworms’ activity over the course of the vermicomposting process. As a result, the end products tended to be more similar in terms of bacterial diversity and richness despite of the initial differences in the community composition of the starting materials (Table 2).

The presence of a core of common bacteria was evidenced in the vermicomposted samples (days 14 to 42) obtained from distilled grape marc (Figure 6). The core microbiome can be defined arbitrarily at any threshold for presence and/or abundance [53]; here we chose the most restrictive option, that is, one ASV should be consistently found in all of the samples from 14–42 days to be considered within the core microbiome regardless of its abundance. We found that the eighty core bacterial taxa clearly exceeded in number the core microbiome (3 ASVs) of raw grape marc [25]. Such a difference in richness may be related to the differences in earthworm density between our study and Kolbe’s [25]. A higher earthworm density is also likely to result in a faster and massive transformation of the fresh substrate into earthworm casts. In fact, some of the core bacterial genera present in the vermicomposted distilled grape marc (e.g., *Mycobacterium*, *Thermomicrobia*, *Rhodobacteraceae*, *Flavobacteriaceae*, *Pseudomonas*, *Chryseobacterium*, and *Acinetobacter*) were also previously found in casts of *E. andrei* [54]. Furthermore, we found in the core microbiome of distilled grape marc members of the phylum Proteobacteria that may contribute to nitrogen fixation (family *Burkholderiaceae* and genus *Devosia* that belongs to the order *Rhizobiales*) and to plant disease suppression (*Pseudomonas*) [55]. *Chitinophagaceae* and other representatives of the phylum Bacteroidetes, which are known to produce a large number of plant cell degrading enzymes with the ability of degrading macromolecules such as chitin and cellulose [56], were also present.

Analysis of functional diversity with PICRUSt revealed that vermicomposting of distilled grape marc resulted in a rapid increase in the metabolic capacity of the bacterial community after 14 days followed by no more significant changes for the duration of the trial (Figure 7). Despite this increase, the functional diversity over the course of the process was higher in the microbiome of raw grape marc than in the microbiome of distilled grape marc (Kolbe et al. [25]; Figure 7 in present study). At the end of the respective trials, the functional diversity in the raw grape marc-derived vermicompost was circa two times higher than that obtained from distilled grape marc (Kolbe et al. [25]; Figure 7 in present study). The strong pressure exerted by certain abiotic factors such as high temperature and low oxygen levels during the process of distillation could have influenced the metabolic capacity of bacterial communities of the fresh distilled grape marc, thereby leading to the subsequent changes throughout the vermicomposting trial.

In addition, processing of distilled grape marc through vermicomposting resulted in increases in the abundance of specific genes involved in the breakdown of cellulose and lignin (Figure 7 inset), and in specific metabolic processes potentially beneficial for plant growth and development, such as the synthesis of salicylic acid (Figure 7 inset). In particular, salicylic acid has been shown to reduce plant stress by inducing plant pathogen resistance mechanisms and increasing the antioxidant activity of plants [57]. However, in contrast to Kolbe et al. [25], vermicomposting of distilled grape marc did not lead to an increase in the genes involved in the biosynthesis of antibiotics (data not shown).

## 5. Conclusions

Proteobacteria and Bacteroidetes dominated the bacterial microbiome over the course of the process as previously shown for raw grape marc, and they contributed with a higher number of ASVs to the core microbiome of distilled grape marc. The compositional changes were accompanied by a rapid increase in richness and taxonomic diversity of bacterial communities on day 14, with values similar to those reported for raw grape marc at the end of the process. Although there was an increase in the metabolic capacity of the bacterial communities after 14 days, the functional diversity of distilled grape marc was lower when compared to raw grape marc. In conclusion, vermicomposting appears as a promising sustainable option to process the valorized distilled grape marc from Albariño grapes, yielding an end product with richer and higher bacterial diversity, and with functional attributes that may enhance the role of distilled grape marc vermicompost for its further use as plant growth promoter and/or soil organic amendment.

## Figures and Tables

**Table 1:** Results from mixed-effects models are shown for α- and β-diversity indices for vermicomposting of distilled grape marc. Significance was determined using analysis of variance (ANOVA). For each test, we report the relevant F statistic (F5,24) and significance (p(> F)). Degrees of freedom were constant across all tests (numerator degrees of freedom: 5; denominator degrees of freedom: 24).

**Alpha Diversity**		**F_5,24_**	**p( > F)**
	Observed	12.004	<0.0001
	Chao1	9.401	<0.0001
	Shannon	54.031	<0.0001
	Faith PD	26.615	<0.0001
**Beta Diversity**		**F_5,24_**	***p*-value**
Unifrac—unweighted	PCoA1	596.074	<0.0001
	PCoA2	26.350	<0.0001
Unifrac—weighted	PCoA1	456.28	<0.0001
	PCoA2	6.101	<0.0001
Bray–Curtis	PCoA1	3629.08	<0.0001
	PCoA2	58.103	<0.0001
Jaccard	PCoA1	2333.82	<0.0001
	PCoA2	70.029	<0.0001

**Table 2:** Comparison of mean α-diversity indices and mean relative abundances of the dominant bacterial phyla between raw and distilled grape marcs at the beginning and the end of the respective vermicomposting trials. Values are given as mean ± standard error. The raw grape marc data were taken from Kolbe et al. [25]

	Day 0		End Process (91 days)	End Process (42 days)
Alpha Diversity	Raw Grape Marc	Distilled Grape Marc	Raw Grape Marc	Distilled Grape Marc
Observed	95.47 ± 7.66	326.26 ± 16.16	685.20 ± 38.87	617.14 ± 50.72
Chao1	100.67 ± 9.54	353.41 ± 17.35	724.43 ± 46.91	637.15 ± 56.05
Shannon	2.40 ± 0.04	4.14 ± 0.07	5.39 ± 0.07	5.29 ± 0.08
Faith PD	10.76 ± 1.16	11.18 ± 0.51	40.62 ± 2.53	27.19 ± 0.52
**Relative Abundances—Phylum**				
Proteobacteria	58.89 ± 2.08	60.01 ± 3.67	52.00 ± 0.95	52.37 ± 1.366
Firmicutes	39.17 ± 2.05	18.87 ± 1.95	0.61 ± 0.05	0.45 ± 0.05
Bacteroidetes	1.09 ± 0.28	14.90 ± 2.82	30.10 ± 1.58	30.29 ± 1.66
Actinobacteria	0.78 ± 0.31	1.34 ± 0.27	9.73 ± 0.94	8.11 ± 0.55
Verrucomicrobia	0.01 ± 0.008	0.08 ± 0.03	3.13 ± 0.23	4.55 ± 0.14
Planctomycetes	0.05 ± 0.02	not detected	2.37 ± 0.20	not detected
Chloroflexi	0.023 ± 0.01	not detected	1.01 ± 0.13	not detected

## Supplementary Materials

The following are available online at https://www.mdpi.com/2076-2607/8/5/642/s1, Figure S1: Rarefaction curves showing the number of amplicon sequence variants (ASV) found in each sample during vermicomposting of distilled grape marc derived from the white winemaking process of the grape variety Albariño. These curves indicate that the sequencing depth was optimal for all of the samples in the full data set. Figure S2: Rarefaction curves showing the number of amplicon sequence variants (ASV) found in each sample during vermicomposting of distilled grape marc derived from the white winemaking process of the grape variety Albariño. These curves indicate that the sequencing depth was optimal for all of the samples in the filtered data set. Figure S3: Boxplots showing the changes in the normalized abundance of bacterial classes of the most abundant phyla during vermicomposting of distilled grape marc derived from the white winemaking process of the grape variety Albariño. Abundance changes are expressed as log2fold. A specific colour is given to the name of the different bacterial classes according to the phylum they belong to. Figure S4: Additional estimates of bacterial α-diversity including A) Chao1 richness and B) Shannon diversity during vermicomposting of distilled grape marc derived from the white winemaking process of the grape variety Albariño. Different letters indicate significant differences between the different stages of the vermicomposting process (Tukey HSD test, FDR corrected). Figure S5: Additional estimates of bacterial β-diversity during vermicomposting of distilled grape marc derived from the white winemaking process of the grape variety Albariño. Principal coordinate analysis with Bray-Curtis (A), weighted UniFrac (B), and Jaccard (C) including days 0, 14, 21, 28, 35 and 42. Principal coordinate analysis with Bray-Curtis (D), weighted UniFrac (E), and Jaccard (F) across days 14, 21, 28, 35 and 42. Different capital and lower case letters indicate significant differences between the different stages of the vermicomposting process in PCoA 1 and PCoA 2 scores, respectively (Tukey HSD test, FDR corrected). Table S1: Shared ASVs between raw and distilled grape marc at the beginning of the vermicomposting trial; Table S2: Shared ASVs between raw and distilled grape marc at the end of the respective vermicomposting trials, that is after 91 and 42 days respectively.
